# Association Between the GGT/HDL-C Ratio and Ultrasonography-Defined NAFLD in Vietnamese Adults: A Cross-Sectional Study

**DOI:** 10.3390/jpm16090441

**Published:** 2026-08-23

**Authors:** Truc Thanh Nguyen, Huong Tu Lam, Thong Duy Vo

**Affiliations:** 1 Endocrinology Department, Tam Anh Ho Chi Minh City General Hospital, Ho Chi Minh City 70000, Vietnam; nguyenthanhtruc91@gmail.com; 2 Department of Internal Medicine, School of Medicine, University of Medicine and Pharmacy at Ho Chi Minh City, Ho Chi Minh City 72714, Vietnam; 3 Department of Gastroenterology, University Medical Center Ho Chi Minh City, Ho Chi Minh City 72714, Vietnam

**Keywords:** non-alcoholic fatty liver disease, gamma-glutamyl transferase, high-density lipoprotein cholesterol, GGT/HDL-C ratio, cardiometabolic risk

## Abstract

**Background and aims.** Nonalcoholic fatty liver disease (NAFLD) is a prevalent chronic liver disease associated with cirrhosis, hepatocellular carcinoma, and increased risks of liver-related and cardiovascular mortality. Many patients, however, remain asymptomatic until advanced stages. The gamma-glutamyl transferase to high-density lipoprotein cholesterol (GGT/HDL-C) ratio is a simple, noninvasive marker reflecting hepatic dysfunction and cardiometabolic imbalance. This study assessed its association with NAFLD and determined an optimal cutoff for identifying NAFLD. **Methods.** We conducted a cross-sectional analysis of 575 adults undergoing routine health examinations at the University of Medicine and Pharmacy Hospital in Ho Chi Minh City between 2021 and 2023. Participants with significant alcohol intake, viral hepatitis, or other chronic liver diseases were excluded. NAFLD was diagnosed by standardized abdominal ultrasonography based on increased hepatic echogenicity relative to the renal cortex or spleen. Associations between GGT/HDL-C and NAFLD were assessed by logistic regression (both as a continuous variable and by quartiles), and diagnostic performance was evaluated using receiver operating characteristic (ROC) analysis. Statistical significance was defined as a two-tailed *p*-value < 0.05. **Results.** Individuals with NAFLD showed significantly higher BMI, prevalence of hypertension and diabetes, and elevated glucose, HbA1c, AST, ALT, GGT, cholesterol, triglycerides, and LDL-C, along with lower HDL-C. Each unit increase in the GGT/HDL-C ratio was associated with a 2% increase in the odds of NAFLD based on univariate logistic regression analysis. However, after adjustment for demographic and metabolic covariates, the GGT/HDL-C ratio was no longer independently associated with NAFLD. The highest quartile had an odds ratio of 7.05 (95% CI: 4.2–11.83) compared to the lowest. The area under the ROC curve for GGT/HDL-C was 0.72, outperforming GGT or HDL-C alone. A cutoff of 32.47 UI/mmol had 72.4% sensitivity and 63.3% specificity. **Conclusions.** The GGT/HDL-C ratio showed a significant unadjusted association with NAFLD and moderate discriminatory performance. However, because the association was no longer significant after full adjustment, the ratio should currently be considered a simple laboratory-based screening marker that requires further validation before routine clinical application.

## 1. Introduction

Although the term metabolic dysfunction-associated steatotic liver disease (MASLD) has recently been proposed to replace non-alcoholic fatty liver disease (NAFLD), the present study used the NAFLD nomenclature because participants were enrolled and diagnosed according to the prevailing diagnostic criteria during the study period. Nevertheless, the findings remain relevant to the evolving MASLD framework because most participants exhibited metabolic risk factors. NAFLD has become the most common chronic liver disorder worldwide, affecting an estimated 25% of the adult population. A five-year longitudinal cohort study in a generally healthy working population (n = 11,448) reported that approximately 12% of individuals developed incident NAFLD as detected by ultrasound over the follow-up period [1]. The disease spectrum ranges from simple hepatic steatosis to non-alcoholic steatohepatitis (NASH), progressive fibrosis, cirrhosis, and hepatocellular carcinoma [2]. Beyond liver-related complications, NAFLD is strongly linked to metabolic disorders such as obesity, insulin resistance, type 2 diabetes, and dyslipidemia [3,4,5]. These comorbidities contribute to a 34–69% higher all-cause mortality over 15 years compared with the general population. Patients with NAFLD also face higher liver-related deaths, increased cardiovascular risk, and an annual 9% rise in hepatocellular carcinoma incidence [6].

Although liver biopsy remains the diagnostic gold standard, its invasiveness and high cost limit its use in routine screening [7,8]. This highlights the need for accessible, noninvasive biomarkers that can facilitate early diagnosis and enable timely intervention.

Gamma-glutamyl transferase (GGT) and high-density lipoprotein cholesterol (HDL-C) have individually been implicated in the pathophysiology of NAFLD, reflecting opposing metabolic processes. GGT, a marker of oxidative stress and hepatic injury, has been strongly correlated with disease severity. For example, Cruz et al. [9] reported that median GGT levels increased significantly with steatosis severity, observing levels of 34.0 UI/L (grade 1), 39.5 UI/L (grade 2), and 57.0 UI/L (grade 3) (*p* = 0.034). In addition, Mansour-Ghanaei et al. [10], Novakovic et al. [11] found elevated GGT to be independently associated with increased NAFLD risk. In contrast, HDL-C, a key anti-inflammatory and antioxidant lipoprotein, is often reduced in individuals with metabolic syndrome and NAFLD [8,12,13,14,15].

Recognizing the complementary pathophysiological relevance of these biomarkers, recent research has proposed the GGT/HDL-C ratio as a more robust indicator for NAFLD. Feng et al. (2020) [16] reported an area under the curve (AUC) of 0.799 (95% CI: 0.788–0.810) for this ratio in a Chinese cohort of 6326 participants. In bariatric surgery patients, the ratio achieved an AUC of 0.81 (95% CI: 0.64–0.98), along with sensitivity and specificity of 82.5% and 77.8%, respectively [17]. Additionally, a large longitudinal cohort of 12,126 non-obese Chinese adults [18] revealed a nonlinear association with new-onset NAFLD, with a saturation threshold estimated at 31.79 UI/mmol.

However, data on the diagnostic performance of this ratio in Southeast Asian populations are still lacking. Unlike composite indices that require multiple anthropometric and biochemical parameters, the GGT/HDL-C ratio integrates two routinely available laboratory markers reflecting complementary pathophysiological pathways—hepatic oxidative stress and dyslipidemia. Its simplicity may make it a potentially useful laboratory marker for preliminary risk assessment, although its incremental value over established steatosis indices remains to be determined. Therefore, this study aimed to investigate the association between the GGT/HDL-C ratio and ultrasonography-defined NAFLD in Vietnamese adults and to explore its discriminatory performance and a cohort-specific cutoff value. The study was not designed to develop or validate a clinical prediction model.

## 2. Materials and Methods

### 2.1. Study Design and Population

This cross-sectional study enrolled adults aged 18 years or older who underwent routine health check-ups at the Health Examination Department of the University Medical Center Ho Chi Minh City between January 2021 and December 2023. Individuals were excluded if they had significant alcohol consumption (>30 g/day for men or >20 g/day for women) [19], viral hepatitis B or C, other chronic liver diseases (including cirrhosis, autoimmune hepatitis, drug-induced hepatitis), pregnancy, or incomplete medical records.

This study was a retrospective analysis of routinely collected clinical data. The study protocol was approved by the Ethics Committee of the University of Medicine and Pharmacy at Ho Chi Minh City (Approval No. 1255/HDDD-DHYD, 14 December 2023). The ethical approval was obtained prior to data extraction and analysis. Given the retrospective nature of the study and the use of de-identified data, informed consent was waived in accordance with institutional regulations and the Declaration of Helsinki.

### 2.2. Data Collection

A total of 1190 medical records were initially reviewed. After exclusion of individuals who met predefined exclusion criteria or had incomplete clinical or laboratory data, 575 participants were eligible for final analysis. Data on anthropometric measurements, medical history, clinical examinations, and laboratory parameters (including AST, ALT, GGT, HDL-C, total cholesterol, triglycerides, and LDL-C) were systematically retrieved from electronic medical records. All blood samples were collected after overnight fasting and analyzed in a single certified laboratory to ensure quality control. Each participant was assigned a unique identification code, and all records were securely stored in the hospital’s centralized database to facilitate consistent data access and verification.

All abdominal ultrasound examinations were performed by two experienced sonographers (each with more than 5 years of experience in abdominal ultrasonography) at the Health Examination Department. Both sonographers held postgraduate qualifications and followed a standardized protocol for the ultrasonographic diagnosis of NAFLD. They were blinded to participants’ clinical and laboratory data to minimize diagnostic bias. All examinations were performed using the same Samsung ultrasound system with identical imaging settings. In addition, regular departmental quality-control procedures and standardized operating protocols were implemented throughout the study period to ensure consistency in image acquisition and interpretation.

### 2.3. Diagnostic Criteria

NAFLD was diagnosed using a combination of imaging findings and exclusion of secondary causes [5]. First, hepatic steatosis was identified by standardized abdominal ultrasonography performed using the same Samsung ultrasound system according to the standardized protocol described above. Diagnosis was based on increased hepatic echogenicity relative to the renal cortex or spleen, with severity categorized as mild (slightly increased echogenicity with clear visualization of intrahepatic vessels), moderate (greater echogenicity partially obscuring vascular structures), or severe (marked echogenicity with significant attenuation of the deep parenchyma). Second, secondary causes of hepatic steatosis were ruled out through clinical history, laboratory evaluation, and medical records, including excessive alcohol consumption, positive hepatitis B surface antigen (HBsAg), positive hepatitis C antibody (anti-HCV), and other chronic liver diseases (including cirrhosis, autoimmune hepatitis, and drug-induced liver disease).

Hypertension was defined according to the ESC/ESH guidelines as systolic blood pressure ≥ 140 mmHg and/or diastolic blood pressure ≥ 90 mmHg, including both previously diagnosed cases under treatment and newly identified cases based on measured values [20]. Diabetes and pre-diabetes were diagnosed according to the criteria of the American Diabetes Association (ADA) 2023, encompassing individuals with established diagnoses, those receiving antidiabetic therapy, and newly detected cases [21].

### 2.4. Statistical Analysis

All analyses were conducted using Stata version 14. Continuous variables were summarized as means with standard deviations or medians with interquartile ranges, depending on distribution assessed by the Kolmogorov–Smirnov test. Categorical variables were presented as counts and percentages. Because BMI followed an approximately normal distribution, it was summarized as mean ± standard deviation (SD), whereas the remaining continuous variables showed non-normal distributions and were presented as median (interquartile range [IQR]).

Group comparisons were performed using chi-square or Fisher’s exact test for categorical variables and independent *t*-tests or Mann–Whitney U tests for continuous variables. Associations between the GGT/HDL-C ratio and NAFLD were evaluated using logistic regression models. To assess the linearity of the association, the GGT/HDL-C ratio was evaluated both as a continuous variable and in quartiles. Diagnostic performance was assessed using receiver operating characteristic (ROC) curve analysis. An exploratory cohort-specific cutoff was identified using the Youden Index, which maximizes the combined sensitivity and specificity, and the Index of Union. This analysis was descriptive and was not intended to establish a validated threshold for clinical use. To assess dose–response relationships, the GGT/HDL-C ratio was additionally analyzed in quartiles, and linear trends were evaluated using the *p*-value for trend. Multivariable logistic regression models were adjusted for demographic (age, sex), metabolic (BMI, diabetes status, HbA1c), hepatic (ALT, AST, GGT), renal (creatinine), and lipid-related variables (HDL-C, LDL-C, triglycerides, total cholesterol). Statistical significance was defined as a two-tailed *p*-value < 0.05. Comparisons between AUROCs were performed using DeLong’s nonparametric test for correlated ROC curves. No clinical prediction model was developed, and no bootstrap validation, cross-validation, or external validation was performed.

## 3. Results

### 3.1. Sample Characteristics

A total of 1190 medical records were reviewed. After applying the eligibility criteria and excluding incomplete records, 575 participants were included in the final analysis. Based on ultrasonographic assessment, 341 (59.3%) participants were classified as having NAFLD and 234 (40.7%) as not having NAFLD. The baseline characteristics of the study participants are presented in Table 1. Compared with individuals without NAFLD, those with NAFLD had significantly higher BMI, prevalence of hypertension and diabetes, and elevated levels of fasting glucose, HbA1c, AST, ALT, GGT, total cholesterol, triglycerides, and LDL-C. In contrast, HDL-C concentrations were significantly lower in the NAFLD group. No significant difference was observed in median age between groups.

### 3.2. Association Between GGT/HDL-C Ratio and NAFLD

Univariate logistic regression analysis revealed a significant positive association between the GGT/HDL-C ratio and the presence of NAFLD. Specifically, for every one-unit increase in the GGT/HDL-C ratio (UI/mmol), the odds of having NAFLD increased by 2% (OR = 1.02; 95% CI: 1.01–1.03; *p* < 0.001). To facilitate clinical interpretation, the GGT/HDL-C ratio was additionally analyzed by quartiles, allowing assessment of the progressive increase in NAFLD risk across clinically relevant categories. The corresponding regression equation was Logit (NAFLD) = 0.02 × (GGT/HDL-C) − 0.59.

To further evaluate this relationship, participants were stratified into quartiles based on GGT/HDL-C ratio values. Compared to the lowest quartile (Q1), the odds of NAFLD were significantly higher in the upper quartiles: OR = 3.25 (95% CI: 2.01–5.27) for Q2, OR = 5.85 (95% CI: 3.50–9.77) for Q3, and OR = 7.05 (95% CI: 4.20–11.83) for Q4 (*p* < 0.001 for trend). These findings indicate a strong dose–response relationship between increasing GGT/HDL-C ratio and NAFLD prevalence, supporting a graded association between increasing GGT/HDL-C ratio and NAFLD prevalence. The univariate quartile-specific estimates are summarized in Table 2.

In the fully adjusted multivariable model, including age, sex, BMI, diabetes status, HbA1c, ALT, AST, GGT, creatinine, HDL-C, LDL-C, triglycerides, and total cholesterol, the GGT/HDL-C ratio was no longer statistically significant. In contrast, several covariates remained independently associated with NAFLD. BMI showed the strongest association (adjusted OR = 1.43; 95% CI: 1.31–1.57; *p* < 0.001). ALT (adjusted OR = 1.04; 95% CI: 1.02–1.07; *p* = 0.001) and HbA1c (adjusted OR = 1.83; 95% CI: 1.41–2.38; *p* < 0.001) were also significant predictors. HDL-C exhibited a borderline inverse association (adjusted OR = 0.32; 95% CI: 0.04–1.07; *p* = 0.059). Other variables—including AST, creatinine, triglycerides, LDL-C, total cholesterol, uric acid, and GGT—were not significant after adjustment.

To evaluate whether the graded association persisted after adjustment, the GGT/HDL-C ratio was additionally modeled in quartiles. Although ORs for higher quartiles remained elevated, the associations were attenuated and no longer statistically significant after full adjustment (Q2 vs. Q1: *p* > 0.05; Q3 vs. Q1: *p* > 0.05; Q4 vs. Q1: *p* > 0.05). This attenuation suggests that the unadjusted relationship between the GGT/HDL-C ratio and NAFLD is partly explained by confounding metabolic factors, particularly BMI, glycemic markers, and liver enzyme levels.

### 3.3. Diagnostic Performance of the GGT/HDL-C Ratio

**Figure 1:**
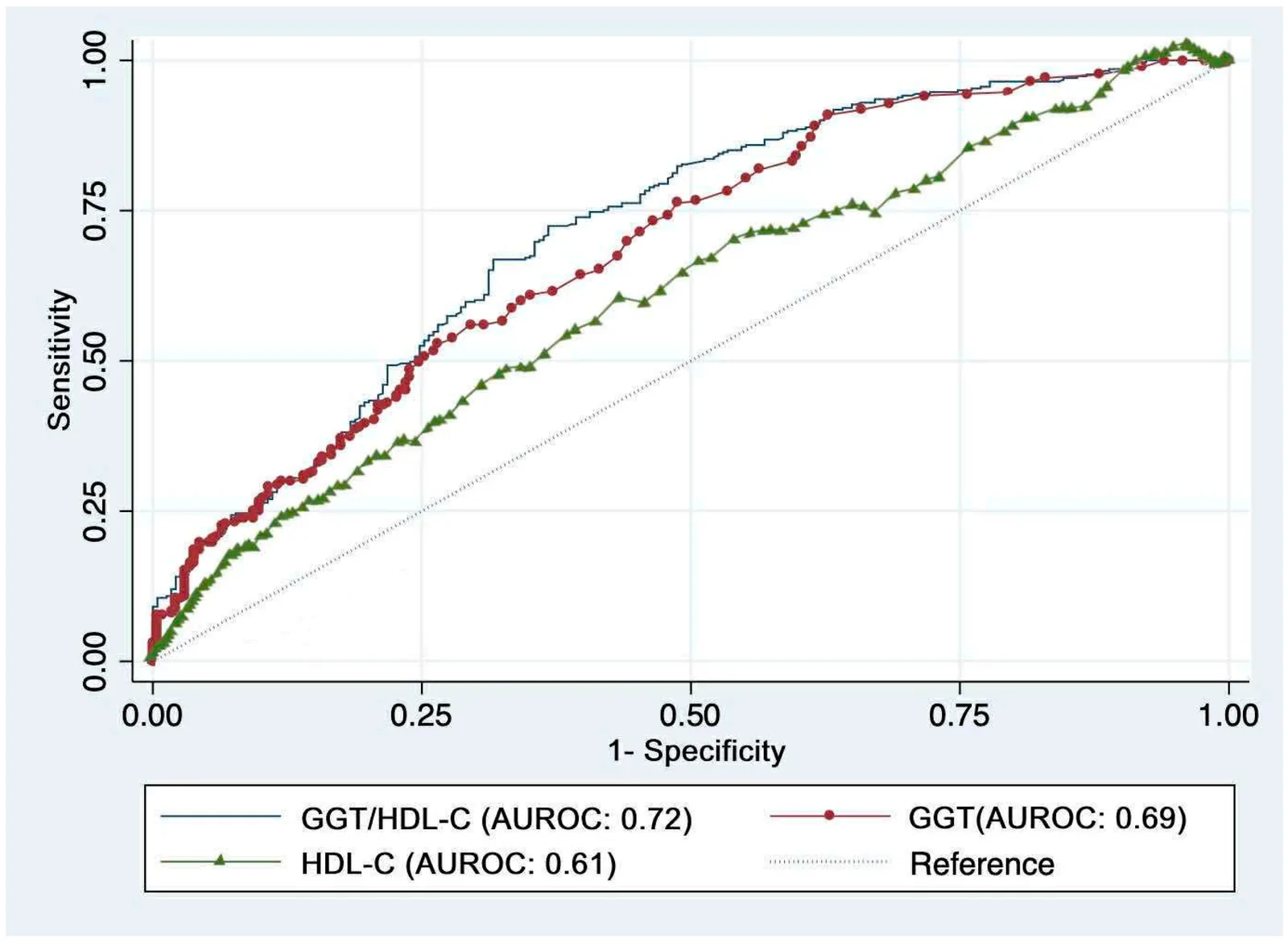
ROC curves for the ability of GGT/HDL-C, GGT, and HDL-C to discriminate the presence of NAFLD.

Receiver operating characteristic (ROC) curve analysis showed that the GGT/HDL-C ratio had an AUROC of 0.72 (95% CI: 0.68–0.76), which was significantly higher than that of GGT alone (AUROC = 0.69) and HDL-C alone (AUROC = 0.61), as confirmed by DeLong’s test (both *p* < 0.05) (Table 3 and Figure 1). In an exploratory analysis, a cohort-specific cutoff of 32.47 IU/mmol corresponded to a sensitivity of 72.4% and a specificity of 63.3% (as shown in Table 4). This cutoff represents apparent performance within the present cohort and should not be interpreted as a validated clinical threshold.

The cutoff value of 32.47 UI/mmol was selected as the optimal threshold because it achieved the highest Youden Index while simultaneously demonstrating the lowest Index of Union, indicating the best balance between sensitivity and specificity.

## 4. Discussion

Our study demonstrated a significant association between the GGT/HDL-C ratio and ultrasonography-defined NAFLD. However, this association was attenuated after adjustment for metabolic confounders, suggesting that the ratio primarily reflects metabolic dysfunction rather than functioning as an independent predictor.

Importantly, although the GGT/HDL-C ratio showed a significant association with NAFLD in the unadjusted analysis, this association was attenuated and no longer statistically significant after adjustment for established metabolic risk factors. This finding suggests that the observed relationship is largely explained by underlying metabolic abnormalities rather than representing an independent predictor of hepatic steatosis. Therefore, the GGT/HDL-C ratio should be interpreted as an integrated laboratory marker reflecting metabolic dysfunction rather than as a standalone diagnostic biomarker. Our findings align with and add to the growing body of evidence supporting the diagnostic value of the GGT/HDL-C ratio for NAFLD. In our Vietnamese cohort, the GGT/HDL-C ratio demonstrated a significantly higher AUROC than either GGT or HDL-C alone, supporting the concept that combining these two routinely available biomarkers improves discrimination compared with either individual marker. Although the AUROC observed in our study (0.72) was lower than that reported by Feng et al. (0.799), both studies consistently demonstrated a positive association between the GGT/HDL-C ratio and NAFLD. The lower discriminative performance observed in our cohort may reflect differences in ethnicity, age distribution, prevalence of metabolic disorders, and study design [16]. Notably, in a Western cohort of obese patients undergoing bariatric surgery, Pajuelo-Vásquez et al. (2024) [17] found an even stronger discriminative ability, with an AUC of 0.81, sensitivity of 82.5%, and specificity of 77.8%. These findings support the reproducibility of the association between the GGT/HDL-C ratio and NAFLD across different populations, although population-specific validation and calibration remain necessary before broader clinical application.

Furthermore, in our analysis, individuals in the highest quartile of the ratio had a 7.05-fold increased odds of NAFLD compared to those in the lowest quartile, reinforcing a clear dose–response relationship similar to the gradient reported by Xie et al. (2022) [18] in a cohort of over 12,000 non-obese Chinese adults. Similarly, the 5-year prospective cohort study of Quiming Li et al. [22] confirmed a significant association (HR = 1.014) between the GGT/HDL-C ratio and NAFLD in non-obese individuals, particularly when the ratio was below 20.35 UI/mmol. Their analysis yielded an AUC of 0.757, further validating its diagnostic relevance.

Our study identified an optimal GGT/HDL-C cutoff of 32.47 UI/mmol (Youden index = 0.35; Index of Union = 0.09), which was higher than the 21.3 UI/mmol reported by Guofang Feng [16], who observed a sensitivity of 74.5% and specificity of 71.7%. This higher cutoff may reflect differences in population characteristics, including age distribution, metabolic comorbidities, assay calibration, and potential ethnic variation across studies. Previous studies by Puukka et al. [23] and Jean-Bernard et al. [24] demonstrated a marked age-related increase in GGT activity. Levels rose by approximately 0.5 UI/L per year between ages 20 and 35, and by up to 64% over a 15-year period, independent of alcohol intake. These age-related variations in GGT levels likely contributed to the higher cutoff observed in our study.

Recent updates in liver disease nomenclature, particularly the transition from NAFLD to MASLD/MAFLD, highlight the central role of metabolic dysfunction in the disease spectrum. The metabolic features reflected by the GGT/HDL-C ratio—such as oxidative stress and lipid–metabolic imbalance—suggest that this marker may remain relevant within the emerging MASLD framework. Although HSI, TyG, and TyG-BMI could potentially be calculated from variables available in the dataset, formal comparison with these indices was not part of the prespecified analytical framework. The present study therefore cannot establish whether the GGT/HDL-C ratio provides superior or incremental discrimination beyond established indices or routinely available clinical variables. Development and validation of comparative prediction models would require a specifically designed analytical framework, including a prespecified base model, calibration assessment, internal validation, and subsequent external validation.

This study has several limitations that should be acknowledged. First, the cross-sectional design prevents any inference of temporal or mechanistic relationships, and therefore the associations observed should not be interpreted as evidence of causality. Second, ultrasonography, while practical, may lack sensitivity for early-stage NAFLD and cannot differentiate between simple steatosis and NASH. Third, the single-center setting and older study population may limit generalizability. In addition, the study did not compare the GGT/HDL-C ratio with established non-invasive NAFLD indices such as the Fatty Liver Index or Hepatic Steatosis Index, which would have provided complementary context for its diagnostic performance. Therefore, the incremental value of the GGT/HDL-C ratio beyond established steatosis indices remains uncertain. Additionally, key metabolic and genetic variables were not evaluated. Further validation in diverse cohorts is warranted. Importantly, the lack of external validation in an independent cohort limits the generalizability and cross-population applicability of our findings. In addition, the proposed cutoff was derived and evaluated in the same cohort, which may have resulted in optimistic estimates of diagnostic performance. Future multicenter studies across different ethnic and clinical settings are warranted to validate population-specific cut-off values.

## 5. Conclusions

The GGT/HDL-C ratio showed a significant unadjusted association and moderate exploratory discriminatory performance for ultrasonography-defined NAFLD but was not independently associated with NAFLD after full adjustment for established metabolic risk factors. The cohort-specific cutoff identified in this study should be considered exploratory and should not be interpreted as a validated clinical threshold. The comparative and incremental value of the GGT/HDL-C ratio beyond established steatosis indices and routinely available clinical variables remains to be determined in appropriately designed studies with internal and external validation.

## Figures and Tables

**Table 1:** Baseline characteristics of participants according to NAFLD status.

Characteristic	General (n = 575)	NAFLD (n = 341)	Non-NAFLD (n = 234)	*p*
Age, years	62 (53–69)	62 (53–70)	62 (50–68)	0.55
Male sex, n (%)	379 (65.9)	244 (71.6)	135 (57.7)	0.001
BMI, kg/m^2^	24.4 (±3.1)	25.6 (±2.8)	22.7 (±2.6)	<0.001
Hypertension, n (%)	382 (66.4)	249 (73)	133 (56.8)	<0.001
Diabetes, n (%)	159 (27.7)	127 (37.2)	32 (13.7)	<0.001
Fasting glucose, mmol/L	5.3 (4.9–6.1)	5.5 (5–6.5)	5.1 (4.8–5.5)	<0.001
HbA1c, %	5.85 (5.5–6.4)	6.04 (5.7–6.8)	5.61 (5.2–6.0)	<0.001
AST, U/L	27 (23–33)	28 (24–36)	26 (23–30)	<0.001
ALT, U/L	25 (17–36)	28 (20–42)	20 (15–28)	<0.001
GGT, U/L	43 (27–79)	53 (34–94)	33 (20–53)	<0.001
Total cholesterol, mmol/L	5.1 (4.3–6.0)	5.2 (4.3–6.1)	5.1 (4.3–5.8)	0.027
HDL-C, mmol/L	1.2 (1.0–1.4)	1.2 (1–1.3)	1.3 (1.1–1.5)	<0.001
LDL-C, mmol/L	3.3 (2.6–3.9)	3.4 (2.7–4.0)	3.3 (2.6–3.9)	0.027
Triglycerides, mmol/L	1.8 (1.3–2.5)	1.97 (1.5–2.9)	1.47 (1.0–2.1)	<0.001
GGT/HDL-C	38 (23–71)	49 (29–86)	25 (16–46)	<0.001

Age is presented in years. Continuous variables are presented as median (interquartile range), except for BMI which is presented as mean ± standard deviation. Categorical variables are shown as number (percentage). All values represent unadjusted descriptive characteristics.

**Table 2:** ORs of GGT/HDL-C ratio quartiles by regression analysis.

Q	GGT/HDL-C Ratio (U/mmol)	OR	95% CI	*p*
1	<21.09	1		
2	21.09–36.03	3.25	2.01–5.27	<0.001
3	36.03–64.65	5.85	3.50–9.77	<0.001
4	≥64.65	7.05	4.20–11.83	<0.001

Odds ratios (ORs) and 95% confidence intervals (CIs) were estimated using univariate logistic regression, with the lowest quartile (Q1) serving as the reference category. Quartiles were defined according to the distribution of the GGT/HDL-C ratio in the study population. A *p* value for trend was calculated by modeling the quartiles as an ordinal variable.

**Table 3:** The area under the ROC curve for the value of GGT/HDL-C, GGT, HDL-C in discriminating the presence of NAFLD.

	AUC	95% CI	*p*
GGT/HDL-C	0.72	0.68–0.76	
GGT	0.69	0.65–0.74	<0.001
HDL	0.61	0.56–0.65	<0.001

**Table 4:** Exploratory cohort-specific cutoff of the GGT/HDL-C ratio for discriminating ultrasonography-defined NAFLD.

Cutoff	Sensitivity	Specificity	Youden Index	Index of Union
30.92	73.9	60.68	0.35	0.13
32.47	72.43	63.25	0.35	0.09
33.02	70.38	63.68	0.34	0.1
34.04	67.45	64.53	0.32	0.12
35.06	66.86	65.38	0.32	0.12

The cutoff and its performance were derived and evaluated in the same cohort without internal or external validation. The estimates should therefore be considered exploratory and may be optimistic.

## Data Availability

The datasets presented in this article are not readily available because they contain de-identified participant-level data subject to institutional data-protection requirements. Requests to access the datasets should be directed to the corresponding author.

## Abbreviations

The following abbreviations are used in this manuscript:

ALT	alanine aminotransferase
AST	aspartate aminotransferase
AUC	area under the curve
BMI	body mass index
CI	confidence interval
GGT	gamma-glutamyl transferase
GGT/HDL-C	gamma-glutamyl transferase to high-density lipoprotein cholesterol ratio
HbA1c	hemoglobin A1c
HDL-C	high-density lipoprotein cholesterol
HCC	hepatocellular carcinoma
HR	hazard ratio
IU	international unit
LDL-C	low-density lipoprotein cholesterol
NAFLD	nonalcoholic fatty liver disease
NASH	nonalcoholic steatohepatitis
OR	odds ratio
ROC	receiver operating characteristic
SD	standard deviation
UI/L	international unit per liter
UI/mmol	unit per millimole
